# Research on Transmission Line Voltage Measurement Method of D-Dot Sensor Based on Gaussian Integral

**DOI:** 10.3390/s18082455

**Published:** 2018-07-28

**Authors:** Jingang Wang, Yanhang Zhao, Wenjiang Li, Xianglong Zeng, Juan Tang, Yao Wang, Xudong Deng

**Affiliations:** 1 State Key Laboratory of Power Transmission Equipment & System Security and New Technology, Chongqing University, Chongqing 400044, China; jingang_023@163.com; 2 Maintenance Branch of State Grid Chongqing Electric Power Company, Chongqing 400039, China; liwenjiang_cqdw@163.com (W.L.); zengxianglong_cqdw@163.com (X.Z.); tangjuan_cqdw@163.com (J.T.); wangyao_cqdw@163.com (Y.W.); dengxudong_cqdw@163.com (X.D.)

**Keywords:** D-dot sensor, electric field inverse problem, transmission line voltage measurement, Gaussian integral

## Abstract

D-dot sensors meet the development trend towards the downsizing, automation and digitalization of voltage sensors and is one of research hotspots for new voltage sensors at present. The traditional voltage measurement system of D-dot sensors makes possible the reverse solving of wire potentials according to the computational principles of the electric field inverse problem by measuring electric field values beneath the transmission line. Nevertheless, as it is limited by the solving method of the electric field inverse problem, the D-dot sensor voltage measurement system is struggling with solving difficulties and poor accuracy. To solve these problems, this paper suggests introducing a Gaussian integral into the D-dot sensor voltage measurement system to accurately measure the voltage of transmission lines. Based on studies of D-dot sensors, a transmission line voltage measurement method based on Gaussian integrals is proposed and used for the simulation of the electric field of a 220 kV and a 20 kV transmission line. The feasibility of the introduction of the Gaussian integral to solve transmission line voltage was verified by the simulation results. Finally, the performance of the Gaussian integral was verified by an experiment using the transmission line voltage measurement platform. The experimental results demonstrated that the D-dot sensor measurement system based on a Gaussian integral achieves high accuracy and the relative error is lower than 0.5%.

## 1. Introduction

D-dot sensors can realize the downsizing, integration and low cost of design due to their simple structure. Moreover, the metal structure is difficult to damage. Therefore, D-dot sensors can satisfy the development needs of the voltage transformers required by intelligent distribution networks in the future and can be widely used in the mature stage of technologies [1,2]. In traditional transmission line voltage measurement using D-dot sensors, the space electric field is measured firstly by the principle of electric field coupling. Secondly, based on the computational principal of the electric field inverse problem, the potentials of the transmission line are acquired by the reverse calculation of the field source parameters according to measured electric field values [3,4,5,6]. However, in the inverse problem operation, the finite element method is usually used to discretize the Poisson equation, and the variable node region needs to be set up. This process may involve the solution of overdetermined equations. Since the electric field information for the whole calculation area cannot be obtained completely, there many problems may occur, such as no solution, multiple solutions, or erroneous solutions. In addition, the different environments will affect the electric field distribution, resulting in difficulty in the calculation of the capacitance matrix, making the calculation difficult. Thus, the voltage solving method using the electromagnetic field inverse problem has some application limitations, as it is limited by the numerical calculation theory and method of the electromagnetic field, as well as the application conditions of the computers involved, and the D-dot sensor voltage measurement system is still challenged by voltage solving difficulties and poor accuracy [7,8,9]. To solve these problems, an integral algorithm is introduced into the D-dot sensor voltage measurement system. A comparison of the Gaussian integral algorithm, Gaussian Legendre integral algorithm, Newton Coates algorithm and other commonly used numerical integration algorithms, shows that the Gaussian integration algorithm has the advantages of high precision, a fast convergence speed and a simple solution process [10,11,12]. Hence, a D-dot sensor transmission line voltage measurement method based on Gaussian integrals is proposed in this paper.

Recently, some experts applied Gaussian integrals to optical voltage sensors and obtained high voltage measurement accuracy [13,14]. However, temperature changes may cause the expansion and concentration of electro-optical crystals due to their material properties, influencing the measurement results [15,16,17]. In addition, optical voltage sensors are a precision instrument and their large-scale application is restricted by their high manufacturing cost and ease of damage during long-distance transportation [18,19,20,21]. Compared with optical methods, D-dot sensors, which have a simple structure, low cost and a wide dynamic range, are easier to use and are more extensively used [14,22]. Therefore, applying Gaussian integrals to the D-dot sensor measurement system, with its simple structure and low cost, will have high research and application values.

The traditional D-dot sensor voltage measurement system reverse calculates transmission line potentials by measuring the space electric field using the electric field inverse problem-solving method [6]. The use of Gaussian integrals can resolve the voltage solving difficulties and poor accuracy of the traditional D-dot sensor voltage measurement system. In the proposed D-dot sensor transmission line voltage measurement method based on Gaussian integrals, the heights of the integral points, that is, the installation positions of the sensors, are calculated by integrating algorithm at first. The field intensity of the integral points is measured using the D-dot sensor measurement system. Finally, the transmission line voltage can be obtained from the weighted sum of the collected signals. 

Firstly, the principle and structure of D-dot sensors are introduced, and the D-dot sensor voltage measurement method of the transmission line based on the Gaussian integral method is proposed. Then, a spatial electric field simulation model of the three-phase transmission line is established, and the simulation results are used to verify the feasibility of the method. Finally, an experimental platform is set up to test the proposed method. The feasibility of the proposed method is proved by the high voltage measurement accuracy of the simulation analysis and experiment. The research results may provide a new direction for the study and development of D-dot sensor transmission line voltage measurement methods.

## 2. The Principle and Structure of D-Dot Sensors

A D-dot sensor is an electronic sensor based on the electric field coupling principle. A D-dot sensor can directly collect electric field information in the calculation area, thereby avoiding contact with the conductor. Moreover, the output signal of a D-dot sensor has a certain proportion to the value of the electric field intensity of the space. By comparing the measured results with the standard electromagnetic field measuring instrument, the ratio between the actual electric field value and the output of sensor can be obtained [1,23]. The electric field intensity of D-dot sensor location is obtained and then the three-phase voltage value can be calculated by using the Gaussian integral algorithm.

**Figure 1:**
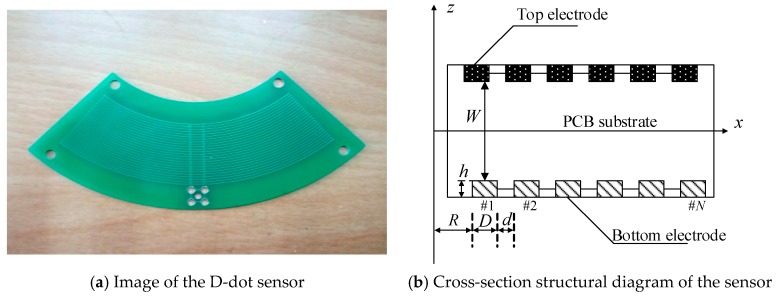
D-dot electric field sensor.

Currently, D-dot sensors have many structural forms. The D-dot sensor used in this experiment is shown in Figure 1a. In difference to the manufacturing methods of the D-dot sensors used in previous studies, the D-dot sensor used in this paper was made in the form of printing circuit board (PCB). It was mainly made of epoxy resin and copper and all the annular electrodes were rectangular cross-sections. The upper and lower layers of PCB were formed by annular electrodes in a series connection. The electrode structure is a concentric arc. These electrodes have different radii, but the same potential. This structure assures that the sensor can perceive the most charges in the space, which increases the sensitivity of the sensors. In addition, the charge induced by the circular arc electrode can be evenly distributed when the sensor is placed in the electric field, and the sensor is protected from damage when the circuit is short-circuited.

A cross-section diagram of the D-dot electric field sensor is shown in Figure 1b. The cross-sections of the electrodes are rectangular and are arranged on the top and bottom of the PCB substrate. The parameters of the D-dot electric field sensor include the distance between the upper and lower two electrodes, *W*; the thickness of the electrode, *h*; the radius of the inner electrode, *R*; the width of the electrode, *D*; and the spacing between the two electrodes, *d*. In the actual fabrication, the spacing of the upper and lower two electrodes, *W,* is 1.6 mm. The detailed structural parameters of the D-dot sensor are shown in Table 1.

## 3. Calculation of the Transmission Line Voltage Based on the Gaussian Integral

### 3.1. Integral Algorithm of Transmission Line Voltage

**Figure 2:**
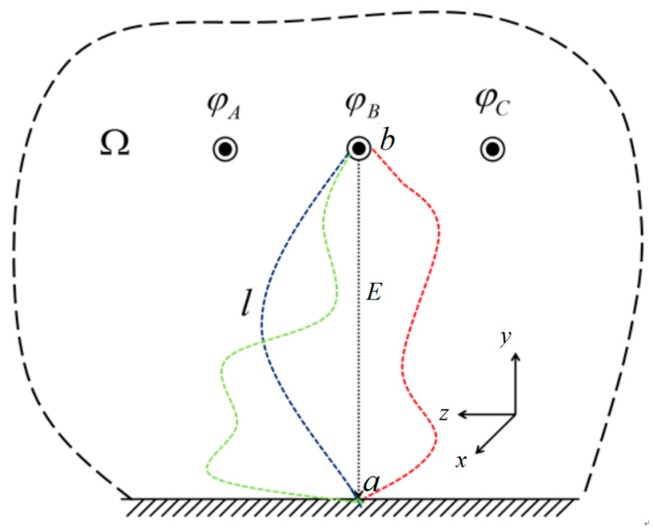
Space electric field composed by three-phase transmission lines.

The power frequency electric field of transmission lines is a quasi-static electric field, and its physical effect can generally be analyzed using the electrostatic field concept. If the ground potential is used as the reference potential, a field-intensity area will form between the transmission lines and grounds (Figure 2). The phase voltage value of a transmission line can be acquired using the integral of the gradient relationship between the electric field intensity and the conductor potential as well as using numerical analysis. Ω represents the solution area of the space electric field. 

If the ground potential is used as the reference potential, the potentials of the three-phase lines are $\varphi_{A}$, $\varphi_{B}$ and $\varphi_{C}$, respectively. An arbitrary integral path *l* from the transmission line to the reference potential is constructed in the computation region (e.g., the red line, blue line or other lines in Figure 2). According to the electromagnetic field theory, the potential difference $U_{ba}$ between any two points (*a* and *b*) along the integral path is correlated with the electric field intensity *E* of the integral path:

(1) $$U_{ba}=-\int_{l}Edl.$$

Under a power frequency of 50 Hz, the electric field can be viewed as a quasi-static field and the electric field intensity is irrotational. Therefore, the potential gap between any two points is unrelated to the integral path [24,25]. For any transmission line (e.g., the B-phase transmission line), the integral path from the transmission line to the ground, which is parallel to the *y*-axis, can be constructed. The direction of the electric field on this path is vertically downward. Therefore, the relationship between the transmission line potential ($\varphi_{B}$) and the electric field intensity on the integral path can be disclosed using the following equation:

(2) $$\varphi_{ba}=-\int_{0}^{b}E_{y}dy,$$

where *E_y_* is a component of the electric field intensity on the integral path in the *y* direction. It can be seen from Equation (2) that $\varphi_{B}$ can be calculated using the linear integral of the electric field intensity on the integral path.

For the measurement of the three-phase transmission line voltages, the three-phase electric field intensity in the computation region is sensitive to the geometric arrangement of the transmission lines and boundary conditions. The electric field intensities on the different integral paths are different. However, the transmission line potential will remain basically the same after changes in the above factors, and the potentials of the different transmission lines are not mutually influential. Similarly, the single-phase transmission line voltage can be calculated using the integral of electric field [26,27].

**Figure 3:**
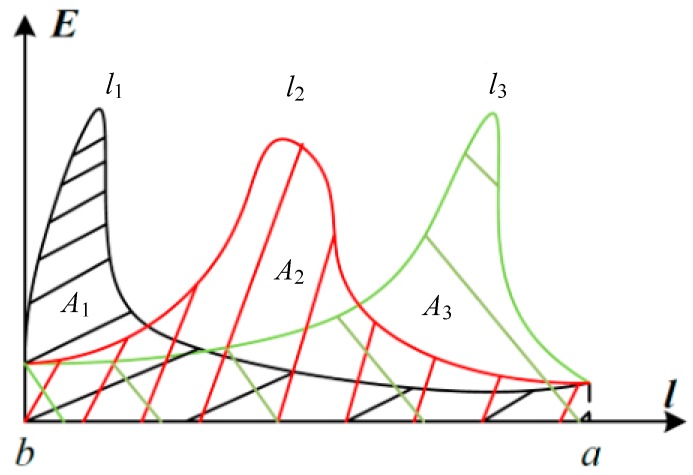
Distribution of the electric field integral of different paths.

In Figure 3, *l*_1_*, l*_2_ and *l*_3_ are three integral paths that were chosen randomly from *b* (transmission line) to *a* (ground reference potential). The horizontal axis represents the position information of the points on the integration path, and the longitudinal axis represents the magnitude of the electric field intensity. The curve is the distribution of the electric field intensity along the integral paths and the area between the curve and the *l*-axis (line integral of the curve to the integral path) is the potential gap between *b* and *a*.

Although the electric field intensity is distributed differently along the three paths, the area between the curves and the *x*-axis must be equal due to the conservation of the electric field (the potential gap is unrelated to the integral paths). Therefore, the effect of the three phase components of the electric field intensity on the integral paths, the shape of the partition curves, the surrounding environment and the electric field intensity changes caused by changes to the geometric position of the three-phase lines can be neglected. Only the integral path from the conductor surface to the potential reference point will be considered. Moreover, the electric field intensity of the integral paths can be measured accurately and the integral results must be potentials on the conductor surface. This property is determined by the single potential value in the conservation field.

### 3.2. Voltage Solving Method Based on Gaussian Integrals

During electric field integration below the transmission line, since each D-dot sensor can only measure electric field intensity on one direction, the *y* direction is chosen as the integral path for the convenience of cooperation between the D-dot sensor and the Gaussian integral. The distribution curve of the electric field along the integral path is a continuous function. However, it is impossible to measure continuous values of electric field intensity in practical applications. As a result, one-dimensional D-dot sensors (along the *y*-axis) were installed at different integral points of the integral path to collect discrete electric field intensities. The electric field at the fixed point of the D-dot sensor is calculated using a numerical integral. The electrical field below the transmission line attenuates exponentially and the computational region is a limited electric field. Hence, a Gaussian integral equation with quick convergence and high accuracy was used as the numerical integral for discrete electric field intensity. For any continuous function *f*(*x*), the general form of Gaussian integration is expressed as:

(3) $$I\left(f\right)=\int_{a}^{b}\rho \left(x\right)f\left(x\right)dx,$$

where ρ(x) is the weight function on the integral interval [*a*,*b*] and ρ(x)≥0 for this integral interval. $I_{n}(f)$ is used to represent the integral formula. $I_{n}(f)$ is used as the approximate expression of I(f). We can then obtain:

(4) $$I_{n}\left(f\right)=\sum_{k=1}^{n}A_{k}f\left(x_{k}\right),$$

(5) $$I\left(f\right)\approx I_{n}\left(f\right).$$

**Figure 4:**
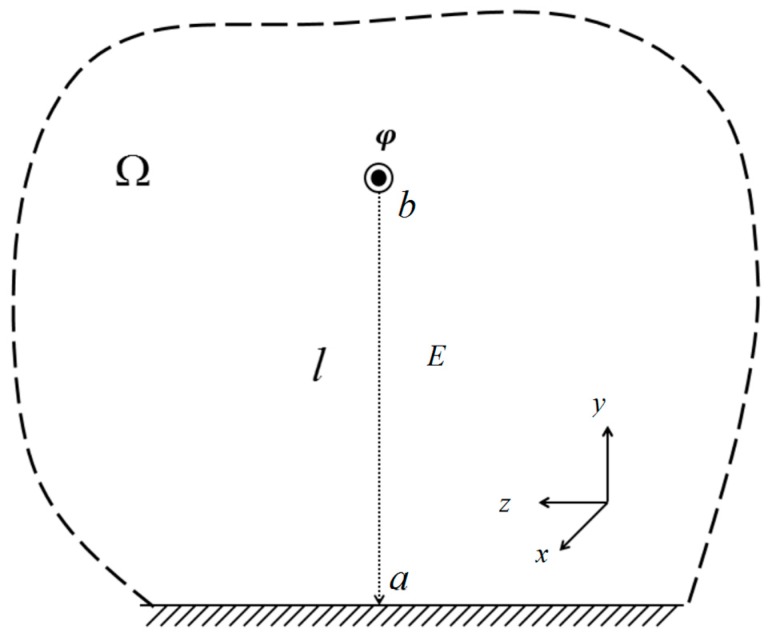
Integral paths.

In the above equation, the integral nodes *x_k_* (*k* = 1,2,3…..*n*) are Gaussian integral points for the interval [*a*,*b*]. $A_{k}$ represents the weighting coefficient of the integral point. When calculating the line voltage based on the electric field integral below the transmission lines, the ground is used as the reference potential and the vertical line below the transmission line is used as an integral path. The integral path is shown in Figure 4.

The electric field integral is equivalent to:

(6) $$U_{ba}=-\int_{0}^{b}E_{x}\left(x\right)dx\approx -\sum_{i=1}^{N}\alpha_{i}E_{x}\left(x_{i}\right),$$

where $E_{x}(x_{i})$ is the electric field intensity at integral point $x_{i}$ and can be measured by the D-dot sensor. $\alpha_{i}$ is the weighting coefficient at the integral sum and *N* is the number of integral nodes. The integral nodes and weighting coefficient can be calculated using specific analysis objects. The testing electric field $E_{x}(x)$ can be described by $E_{x}(x)=\rho (x)E_{x}^{unp}(x)$, where ρ(x) is the weight function and $E_{x}^{unp}(x)$ is the electric field intensity at free disturbance.

Let $\alpha_{i}=\beta_{i}/E_{x}^{unp}\left(x_{i}\right)$. $\beta_{i}$ is an intermediate variable introduced in order to make the calculation process clearer and more intuitive. The formula is introduced into Equation (6):

(7) $$-\int_{0}^{b}E_{x}^{unp}\left(x\right)\rho \left(x\right)dx\approx -\sum_{i=1}^{N}\beta_{i}\rho \left(x_{i}\right).$$

The weight function ρ(x) is a series of incremental polynomials to assure the accuracy of the results of Equation (7). Considering that there are *2n* unknown quantities to be solved ($\beta_{1},\beta_{2}\cdots \beta_{N},x_{1},x_{2}\cdots x_{N}$), it is determined that $\rho (x)=1,x,x^{2},x^{3},\cdots ,x^{2N-1}$; thus the following formula can be obtained:

(8) $$\left\{ \begin{array}{l} m_{0}=\beta_{1}+\beta_{2}+\cdots +\beta_{N}\\ m_{1}=\beta_{1}x_{1}+\beta_{2}x_{2}+\cdots +\beta_{N}x_{N}\\ m_{2}=\beta_{1}x_{1}^{2}+\beta_{2}x_{2}^{2}+\cdots +\beta_{N}x_{N}^{2}\\ \cdots \cdots \\ m_{2N-1}=\beta_{1}x_{1}^{2N-1}+\beta_{2}x_{2}^{2N-1}+\cdots +\beta_{N}x_{N}^{2N-1} \end{array}\right.,$$

(9) $$m_{k}=\int_{0}^{b}E_{x}^{unp}\left(x\right)x^{k}dx.$$

The $m_{k}$ is used to represent the integral result of Formula (9). The characteristic polynomial coefficient of *x* can be obtained from the mathematic operation of Equations (8) and (9), thus enabling the calculation of $x_{i}$. The three solutions of the polynomial equations are $x_{1}$, $x_{2}$, and $x_{3}$, which is the height of three integral points. $\beta_{i}$ can be calculated by any *N* equations in Equation (8). Finally, the desired $\alpha_{i}$ and $x_{i}$ are determined. This algorithm can be realized using MATLAB programming. Part of the MATLAB code is shown in Appendix A. In actual applications, the D-dot sensor only has to measure the electric field intensity at integral points. On this basis, the potential of the transmission lines ($U_{ba}$) can be calculated using the Gaussian integral algorithm.

### 3.3. Solving Transmission Line Voltage

**Figure 5:**
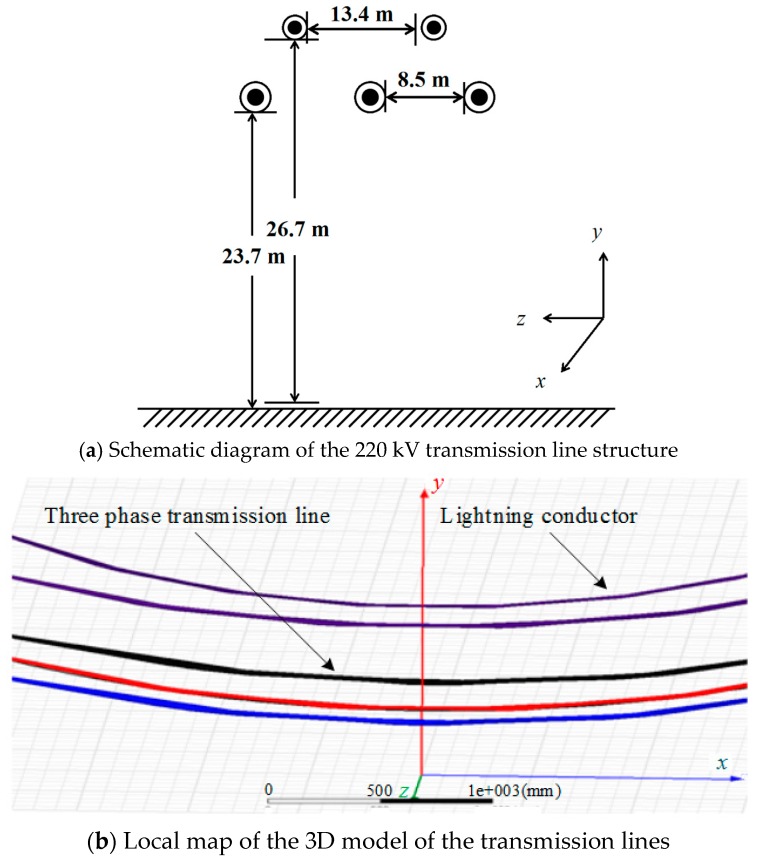
The 220 kV three-phase transmission line simulation model.

The voltage level that is extensively used in transmission lines is 220 kV. Here, the voltage of 220 kV transmission lines was calculated using the electric field integral method. A simulation model of electric field distribution below the transmission lines was constructed using the simulation tool Ansoft. According to the actual working conditions of transmission lines, three lines were distributed horizontally at equal intervals. The phase interval was 8.5 m and the transmission lines were 23.7 m above the ground. The horizontal interval between the two lightning conductors in the upper position was 13.4 m. They were 26.7 m above the ground and the span was set to 220 m. Figure 5a is a structural diagram of the transmission line. The 3D transmission line model which was constructed using the Ansoft Maxwell simulation software is shown in Figure 5b. A rectangular coordinate system was used and the ground was viewed as an infinitely large plane with one potential.

**Figure 6:**
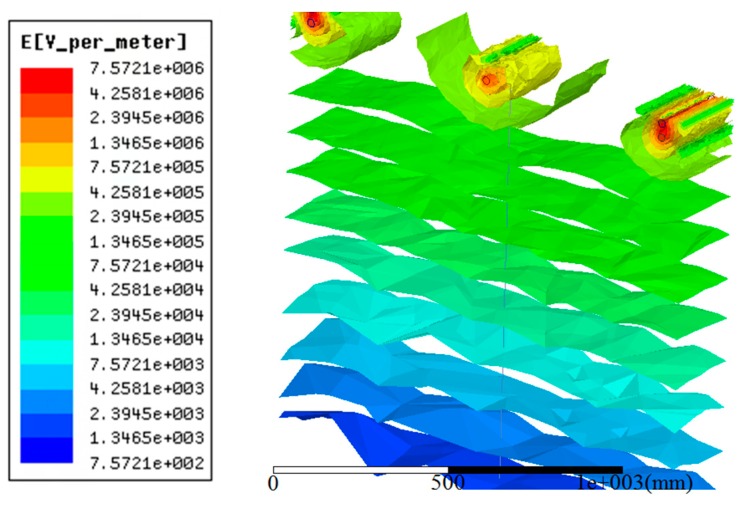
Electric field distribution of the 220 kV transmission lines.

The 3D distribution of the electric field intensity surrounding the 220 kV transmission lines is shown Figure 6.

**Figure 7:**
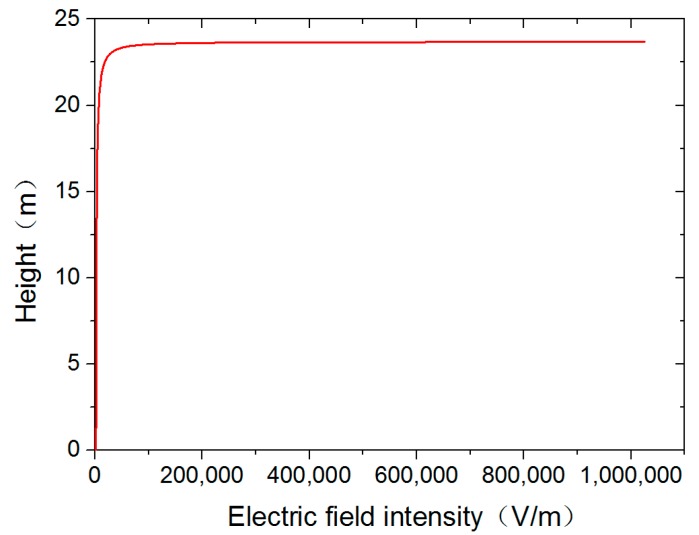
Electric field distribution curve under the 220 kV transmission line.

The electric field data under the conductor was obtained through a simulation. The electric field data were imported into the Origin software and the electric field distribution curve in Figure 7 was produced. The longitudinal axis in the picture indicates the height of the position from the ground and the horizontal axis represents the magnitude of the electric field at that location. The intuitive electric field distribution can be seen in Figure 7 and the electric field values at specific positions can be found.

**Figure 8:**
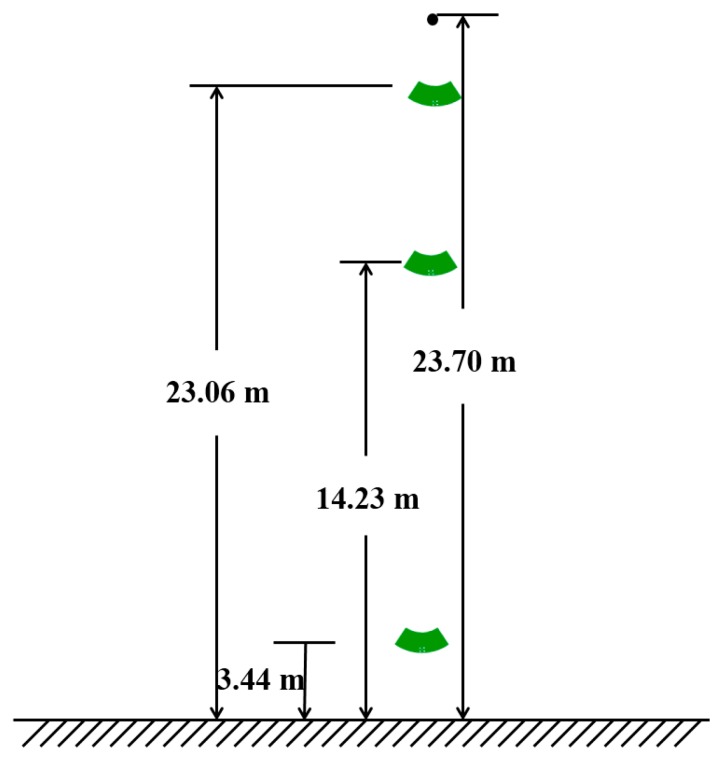
Schematic of the installation location of the 220 kV transmission line sensors.

According to the characteristics of the Gaussian integral algorithm, the accuracy of the solution increases as the number of integral points increases. The two point integration algorithm has only first order algebraic accuracy and struggles to meet the needs of grid voltage measurement [28,29]. The three point integral algorithm and the four point integral algorithm have higher accuracy and can meet the accuracy requirements of voltage measurement. Considering the practicability of the measurement method, the increase of integral points will increase the difficulty of the installation of the measuring system. Therefore, this paper takes three Gaussian integral points and uses the simulation results from Figure 6, combined with Formulae (8) and (9), to calculate the three points of the 220 kV transmission line. The three integral point coordinates are as follows: 23.06 m, 14.23 m and 3.44 m. The D-dot sensor installation location schematic is provided in Figure 8.

According to the calculated integral point coordinates *x_i_*, the field strength $E_{x_{i}}$ of the integral point can be obtained as in Figure 6. Combined with Formulae (6)–(8), the integral point weight $\alpha_{i}$ and integral result *Us* can be obtained, combining the integral result *Us* and the phase voltage *U* of the 220 kV transmission line. The relative error ε of the integral result was calculated according to the following formula:

(10) $$\varepsilon =\frac{\left(U-U_{s}\right)}{U}\times 100\%$$

Finally, the Gaussian integral method was used to obtain the phase voltage amplitude of the 220 kV transmission line, which is 179.94 kV. The detailed results and relative errors are presented in Table 2.

As can be seen from Table 1, the field intensities of the integral points were simulated values under the 220 kV voltage level. When three integral points are used, the Gaussian integral can calculate the integral voltage accurately, showing a relative error of 0.17%.

In order to further verify the accuracy of the D-dot sensor voltage measurement method for transmission lines based on the Gaussian integral method, it is convenient to test and verify the simulation results. According to the above method, a simulation model for a 20 kV three-phase transmission line was established, and the electric field distribution under the 20 kV transmission line was simulated and analyzed. The transmission line was a copper wire of 1 cm diameter, and the distance between the conductor and the ground was 1.5 m, and the distance between the wires was 0.6 m. Finally, the Gaussian integral algorithm was used to solve the simulation results of the 20 kV transmission lines, and the relative error of the solution results was obtained by using Formula (10). The integral point coordinates *x_i_*, the integral point field strength $E_{x_{i}}$, the integral point weight $\alpha_{i}$, the integral result *Us* and relative error ε are shown in Table 3.

As shown in Table 2 and Table 3, according to the simulation results for the electric field distribution under the two voltage transmission lines of 220 kV and 20 kV, it was found that when three integral points are taken, the two voltages are highly accurate when using the Gaussian integral method, and the relative error of the results obtained by the simulation data was less than 0.17%.

## 4. Experiment Analysis and Verification

**Figure 9:**
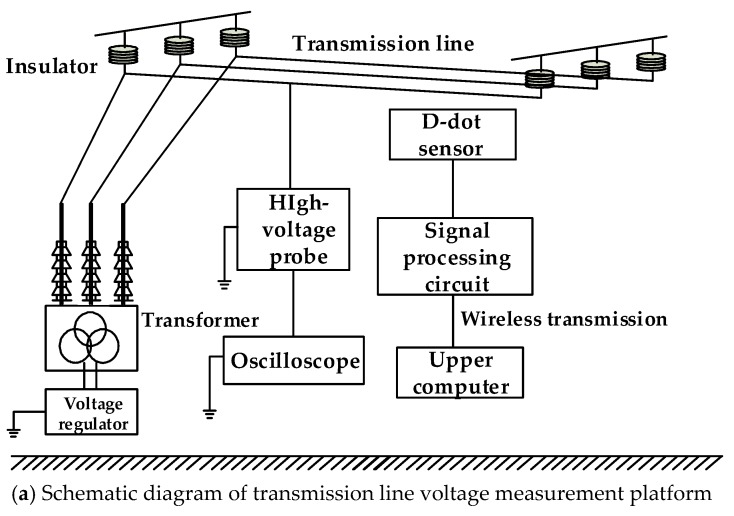

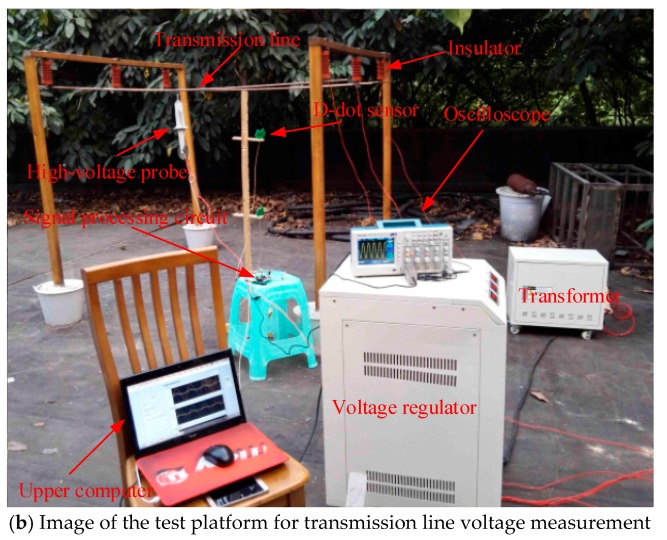
Experimental platform for the voltage measurement of the transmission line.

To verify the accuracy of the Gaussian integral in the D-dot sensor transmission line voltage measurement system, a simulation analysis and experiment was carried out for 5 kV, 10 kV, 15 kV and 20 kV transmission lines. Firstly, the electric field distribution below the transmission lines was simulated. The coordinates and weight of the integral points were calculated using the three-point Gaussian integral. Finally, the test platform was constructed. The electric field values at the integral points were measured by D-dot sensor and the transmission line voltage was solved using a Gaussian integral. In actual working conditions, the accuracy of the Gaussian integral in calculating the transmission line voltage was tested. The test platform for the three-phase transmission line voltage measurement is shown in Figure 9.

As shown in Figure 9a, the three-phase voltage regulator and the step-up transformer generate a controllable three-phase power frequency voltage as the power supply part of the experimental platform. The voltage of the transmission line is measured by the TEC P6015A high-voltage probe (Tektronix (China) Co. Ltd., Shanghai, China). The attenuation ratio of the high-voltage probe is 1000:1, and it has a phase compensation circuit, which is used as the actual voltage of the wire after correction. The D-dot voltage measurement system is composed of three parts: the D-dot sensor, signal processing circuit and upper computer. In the upper computer, the Gaussian integration algorithm is used to deal with the electric field information measured by the sensor. Figure 9b is a physical diagram of a three-phase transmission line voltage measurement test platform. The transmission wire used was copper wire with a diameter of 1 cm. The distance between the wire and ground was 1.5 m, and the distance between the lines was 0.6 m.

**Figure 10:**
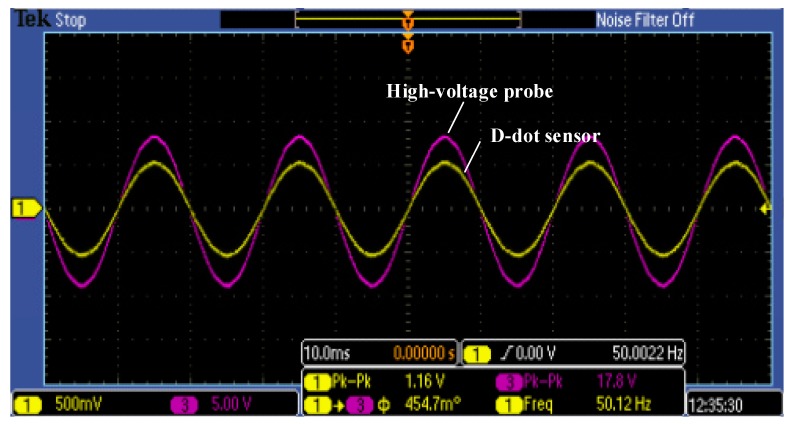
Comparison of outputs between the D-dot sensor and high-voltage probe.

The oscilloscope collected the outputs of the high-voltage probe and the top sensor at the same time to verify measurement effect of the sensor. The output of the high-voltage probe was used as the standard voltage. The results displayed by the oscilloscope are presented in Figure 10.

**Figure 11:**
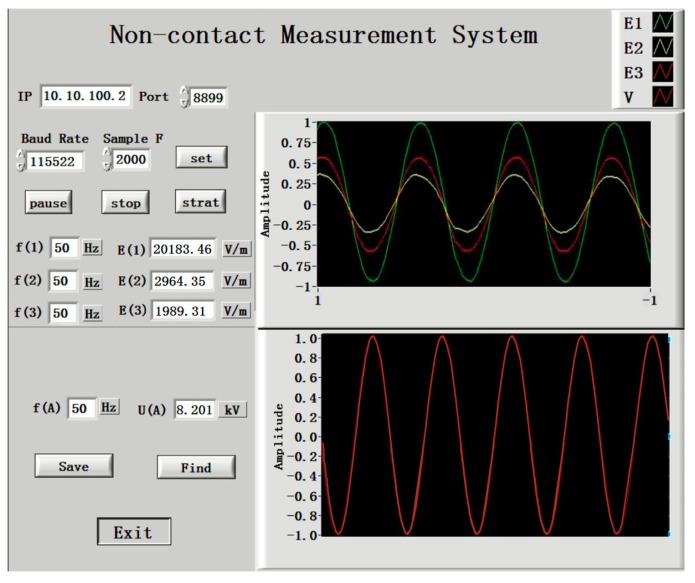
The front panel of the voltage measurement system.

Three D-dot sensors were placed at integral points below the transmission line. They collected electric field information synchronously and converted the information into discrete electric field signals using the hardware circuit. These discrete signals were sent to LabVIEW on the upper computer by the wireless module and were then displayed in the front panel after the signal processing module. The designed front panel is shown in Figure 11. The data sampling frequency can be set using the front panel. After test starts, the front panel displays the electric field value at the three integral points and the actual voltage values calculated using the Gaussian integral. The electric field waveform and transmission line voltage waveform that were detected by sensor are displayed.

The maximum voltage that was produced by the three-phase regulator and boosting transformer in the test platform was 20 kV. Hence, 5 kV, 10 kV, 15 kV and 20 kV transmission lines were tested on the test platform. The position of the sensor *x_i_* and the weight of the integral point $\alpha_{i}$ can be calculated using the Gaussian algorithm. The D-dot sensor was used to measure the field strength $E_{x_{i}}$ of the integral point, and the numerical integration voltage *Us* can be obtained by the weighted summation of the field strength of the integration point. The actual relative error ε can be obtained by including the value *Us* of numerical integral voltage value and the actual voltage *U* measured by high-voltage probe in Formula (10). The detailed results and relative errors are presented in Table 4.

The electric field distribution below the transmission lines was simulated and the installation positions of the D-dot sensors were calculated using the Gaussian integral. At the same time, the actual voltage was calculated from the weighted sum of the electric field values detected by the sensors. By comparing the simulation and test results for the 20 kV transmission lines presented in Table 3 and Table 4, it was found that in both the simulation and the test the use of the Gaussian integral method to solve the line voltage of the D-dot voltage measurement system was highly accurate. Moreover, the accuracy of the simulation results was higher than that of the experimental results, which may have been caused by the installation location and site interference. By comparing the voltage calculated by the Gaussian integral with the voltage measured by the high-voltage probe, it can be seen that the Gaussian integral method can effectively find the transmission line voltage, and the relative error of the voltage solution is less than 0.5%.

## 5. Conclusions and Future Work

Based on studies of D-dot sensors, this paper suggested introducing a Gaussian integral into the D-dot sensor voltage measurement system and proposed a transmission line voltage measurement method based on Gaussian integrals. In the proposed method, the positions of three integral nodes were calculated using Gaussian integrals. Later, the electric field values at integral points were measured using D-dot sensors. Finally, the transmission line voltage was calculated from the weighted sum of the detected electric field values. Some major conclusions were able to be drawn:

(1) A transmission model of the 220 kV and 20 kV transmission lines was constructed, which disclosed the electric field distribution below the transmission lines. The transmission line voltage was solved using a Gaussian integral and the relative error of the result was less than 0.17%.

(2) An electric field measurement platform of transmission lines was constructed to test the performance of the proposed method. The results demonstrate that the error of the proposed method is lower than 0.5%, meeting the voltage measurement requirements of power grids.

(3) The proposed method enjoys promising application prospects in voltage monitoring, electric energy measurement, and relay protection, due to the high measurement accuracy and simple solving process. However, further studies on multiple aspects are needed to solve existing problems, such as sensor installation difficulties and the high sensitivity of the measurement accuracy to installation points. Future work must determine whether the required precision of the sensor installation can be reduced by optimizing the integration algorithm and how this method can be extended to the measurement of three-phase voltage. 

## Figures and Tables

**Table 1:** Main structural parameters of the D-dot electric field sensor.

Parameter	Number	*R*/mm	*h*/mm	*D*/mm	*d*/mm
Top electrode	15	44	0.035	0.1524	0.1524
Bottom electrode	12	49.08	0.035	0.254	0.254

**Table 2:** Integration results of the Gaussian algorithm for the 220 kV transmission line.

Integral Point Coordinates (m)	Integral Point Field Intensity (V/m)	Integral Point Weight	Numerical Integral Voltage (kV)	Actual Voltage (kV)	Relative Error
23.06	31,028.60	3.9138	179.94	179.63	0.17%
14.23	3383.27	11.9956			
3.44	2430.33	7.3718			

**Table 3:** Integration results of the Gaussian algorithm for 20 kV transmission lines.

Integral Point Coordinates (m)	Integral Point Field Intensity (V/m)	Integral Point Weight	Numerical Integral Voltage (kV)	Actual Voltage (kV)	Relative Error
1.44	40,370.27	0.2505	16.353	16.330	0.14%
0.90	5946.42	0.7396			
0.22	3975.56	0.4634			

**Table 4:** The results of the Gaussian integral from the experiment.

Valid Value of Line Voltage (kV)	Integral Point Coordinates (m)	Integral Point Field Intensity (V/m)	Integral Point Weight	Numerical Integral Voltage (kV)	High-Voltage Probe (kV)	Relative Error
5	1.44	10,095.43	0.2516	4.102	4.09	0.31%
	0.90	1482.72	0.7425			
	0.22	995.02	0.4635			
10	1.44	20,183.46	0.2516	8.201	8.18	0.25%
	0.90	2964.35	0.7426			
	0.22	1989.31	0.4635			
15	1.44	30,138.90	0.2530	12.33	12.3	0.27%
	0.90	4439.03	0.7443			
	0.22	2999.42	0.4667			
20	1.44	40,560.46	0.2505	16.43	16.4	0.18%
	0.90	5971.06	0.7396			
	0.22	3995.73	0.4634			

## Appendix A. Matlab Code

syms c0 c1 c2 x;

[c0,c1,c2]=solve(‘c0*m0+c1*m0+c2*m2+m3=0′,’c0*m1+c1*m2+c2*m3+m4=0′,’c0*m2+c1*m3+c2*m4+m5=0′); % The solution of the coefficient of the characteristic equation.

c0=subs(c0);

c1=subs(c1);

c2=subs(c2);

f=c0+c1*x+c2*x^2+x^3;

p=sym2poly(f);

x=roots(p);% The solution the position of three integral points.

x1=x(1);

x2=x(2);

x3=x(3);

[beta1,beta2,beta3]=solve(‘beta1+beta2+beta3-m0=0′,’x1*beta1+x2*beta2+x3*beta3-m1=0′,’(x1^2)*(beta1)+(x2^2)*(beta2)+(x3^2)*(beta3)-a=0′);

beta1=subs(beta1);

beta2=subs(beta2);

beta3=subs(beta3);

beta=[beta1; beta2; beta3]

alpha=beta./Ex;% The solution of the weight of the integral point.

alpha1=subs(alpha(1))

alpha2=subs(alpha(2))

alpha3=subs(alpha(3))
